# Immunotherapy for Microsatellite-Stable Metastatic Colorectal Cancer: Can we close the Gap between Potential and Practice?

**DOI:** 10.1007/s11912-024-01583-w

**Published:** 2024-07-30

**Authors:** Marwa Abdel Hamid, Lorenz M. Pammer, Theresa K. Lentner, Bernhard Doleschal, Rebecca Gruber, Florian Kocher, Elisabeth Gasser, Anna Jöbstl, Andreas Seeber, Arno Amann

**Affiliations:** 1 Department of Hematology and Oncology, Comprehensive Cancer Center Innsbruck, Medical University of Innsbruck, Anichstrasse 35, 6020 Innsbruck, Austria; 2 Department of Gastroenterology and Hepatology, Medical University of Innsbruck, Innsbruck, Austria; 3 Clinical Department for Internal Medicine, University Hospital St. Poelten, Karl Landsteiner University of Health Sciences, St. Poelten, Austria; 4 Department of Internal Medicine I for Hematology With Stem Cell Transplantation, Hemostaseology and Medical Oncology, Ordensklinikum Linz, Linz, Austria; 5 Medical Faculty, Johannes Kepler University Linz, Linz, Austria; 6 Department of Visceral, Transplant and Thoracic Surgery, Center of Operative Medicine, Medical University of Innsbruck, Innsbruck, Austria; 7 Department of Radiology, Medical University of Innsbruck, Innsbruck, Austria

**Keywords:** Colorectal cancer, Immunotherapy, Biomarker, Microsatellite stable, Microsatellite instability

## Abstract

**Purpose of Review:**

This review will explore various strategies to rendering MSS mCRCs susceptible to ICI. Moreover, we will provide an overview of potential biomarkers that may aid to better patient selection, and discuss ongoing efforts in this area of research.

**Recent Findings:**

Colorectal cancer (CRC) ranks among the top three most common cancers worldwide. While significant advances in treatment strategies have improved the prognosis for patients in the early stages of the disease, treatment options for metastatic CRC (mCRC) remain limited. Although immune checkpoint inhibitors (ICI) have revolutionized the treatment of several malignancies, its efficacy in mCRC is largely confined to patients exhibiting a high microsatellite instability status (MSI-H). However, the vast majority of mCRC patients do not exhibit a MSI-H, but are microsatellite stable (MSS). In these patients ICIs are largely ineffective.

**Summary:**

So far, ICIs do not play a crucial role in patients with MSS mCRC, despite the promising data for inducing long-term remissions in other tumour entities. For this reason, novel treatment strategies are needed to overcome the primary resistance upon ICI in patients with MSS.

## Introduction

Colorectal cancer (CRC) is the third most common cancer globally, and represents the second most common malignant tumor in women and the third most common in men [1]. The prognosis of patients with CRC depends on the stage of disease at initial diagnosis and other clinical and biological risk factors [2]. Regrettably, around 50% of patients are diagnosed at an advanced stage initially [3]. Although great advances in the treatment of advanced or metastasized CRC (mCRC) have been achieved, it remains one of the leading causes of death worldwide [2].

In recent decades, a significant wave of approved targeted therapeutics has been integrated into clinical practice. Understanding molecular signaling networks that control intestinal regeneration and homeostasis has helped to discover key drivers in CRC pathogenesis, progression and novel treatment targets [4]. Molecular profiling and tumor characterization, including chromosomal instability, microsatellite instability (MSI), and the CpG island methylator phenotype, have thus, become essential tools in guiding clinical decision making [3]. While some novel therapies, such as drugs targeting *KRAS* [5] or *BRAF* [6] mutations, have shown outstanding clinical improvement, other strategies such as immune checkpoint inhibition (ICI) has only proven to be effective in patients with mCRC that are mismatch-repair-deficient (dMMR) or MSI-High (MSI-H) mCRC [3]. One of the first trials, investigating the efficacy of ICI in dMMR/MSI-H CRCs was published in 2015. Le et al*.* directed a phase 2 trial aiming to investigate the efficacy of the anti-PD1 checkpoint inhibitor pembrolizumab in patients with mCRC with or without dMMR. They observed that a high somatic mutational load was related to a longer progression-free survival (PFS) (p = 0.02) when treated with ICI, and thus concluded that MMR status might be predict benefit of ICI [7]. The subsequent phase 3 Keynote-177 trial was able to demonstrate a meaningful superiority in terms of response rates, PFS and overall survival (OS) of pembrolizumab in comparison to standard-of-care chemoimmunotherapy, which finally led to the approval of pembrolizumab as a new first-line therapy in mCRC patients with dMMR/MSI-H mCRC [8]. Similar promising results were observed with another anti-PD1 inhibitor (nivolumab), which was primarily investigated in the phase 2 CheckMate-142 trial [9]. The combination of nivolumab and the anti cytotoxic T-lymphocyte-associated protein (CTLA4) inhibitor ipilimumab improved even the results of nivolumab monotherapy [10, 11] in terms of prolonging survival leading to its approval as a second-line treatment option in patients with dMMR/MSI-H mCRC [3]. The subsequent phase 3 CheckMate-8HW trial evaluated the combination of nivolumab and ipilimumab in the first-line setting in mCRCs. The trial met its primary endpoint in showing a prolongation of PFS in the ICI cohort in comparison to standard-of-care chemoimmunotherapy [12]. For this reason, nivolumab plus ipilimumab represent a further first-line treatment option in patients with dMMR/MSI-H mCRC next to pembrolizumab monotherapy.

Although ICI provides a clinically effective and long lasting anti-cancerous response, the majority of mCRC patients are mismatch-repair-proficient (pMMR), microsatellite-stable (MSS), or have low MSI (MSI-L) status, and thus do not benefit from this treatment highlighting the strong need for alternative strategies. The mechanisms of resistance in these tumors, also referred to as “cold” tumors, is an ongoing topic of research. Especially the idea of ICI-based combinational treatments to boost sensitivity to ICI is of great clinical interest [13]. This review will provide a landscape of various current strategies to overcome resistance to ICI and how to change the treatment paradigm in pMMR/MSS mCRC.

### Exploring Innovative Treatment Modalities Based on the Latest Research Results

Recent clinical trials have made progress in exploring ICI options for dMMR/MSS mCRC. Table 1 summarises the most recent studies (ongoing, completed and discontinued studies) presented at the most recent oncology conferences in 2023 and 2024. In the following sections several of the listed options will be discussed more in detail.

**Table 1:** Summary of clinical trials investigating immune checkpoint inhibitors ins microsatellite-stable colorectal cancers

Clinical trial identifier/study title	Year	Design	Treatment line	Treatment regimen	Number of patients	ORR	mPFS (months)	mOS (months)	Efficacy	Additional comments
			**IO + Chemo**							
NCT02848443	2021	1	≥ 1 prior line of treatment	Trifluridine/tipiracil + Oxaliplatin + Nivolumab or Bevacizumab	17	7,1%	6	NA	NA	
NCT02860546	2021	2	≥ 2 previous lines of chemotherapy	Trifluridine/tipiracil + Nivolumab	18	0%	2,8	NA	NA	
NCT05171660	2022	2	First line	Sintilimab + CAPOX + Bevacizumab	25	84%	NA	NA	NA	RAS mt + MSS; ORR lung lesions: 100%; ORR liver lesions: 93,3%
NCT02873195/BACCI	2022	2	≥ 2 previous lines of chemotherapy	Capecitabine + Bevacizumab + Atezolizumab	46	4,4%	3,3	NA	PFS: HR 0,82; p = 0,155 vs Capecitabin + Bevacizumab	Higher response rate and OS in patients without liver metastases in the atezolizumab arm (ORR 23.1 vs. 5.8%, HR for OS 3.33 vs. 1.14)
NCT03202758/MEDITREME	2023	1/2	First line	mFOLFOX6 + Durvalumab + Tremelimumab	57	61%	8,4	NA	NA	RAS mt + MSS
NCT03721653/AtezoTRIBE	2023	2	First line	FOLFOXIRI + Bevacizumab + Atezolizumab	145	64%	13	30,8	PFS: HR 0.79; p = 0,074 OS: HR 0,83; p = 0,172 vs FOLFOXIRI + Bevacizumab	IS IC high and TMB high derive greater benefit
NCT04660812	2024	2	2nd line	Etrumadenant + Zimberelimab + FOLFOX + Bevacizumab	112	13%	6,2	19,7	PFS: HR 0.27; p = 0.0001) OS: HR 0.37; p = 0.0003) vs Regorafenib	
NCT03414983/Checkmate 9 × 8	2024	2	First line	Nivolumab + mFOLFOX6 + Bevacizumab	127	60%	11,9	29,2	PFS; HR 0.81; p = 0.30	
NCT03539822/MAYA	2022	2	≥ 2 previous lines of chemotherapy	Temozolomid + Nivolumab + Ipilimumab	135	45%	7,1	18,5		MGMT silenced
			**IO + TKI**							
NCT03406871/REGONIVO/EP01603	2020	1/2	≤ 2 previous lines of chemotherapy	Regorafenib + Nivolumab	25	0,3	NA	NA	NA	ORR lung lesions: 63.6%, ORR liver lesions: 8.3%
NCT03712943	2022	1	≥ 2 previous lines of chemotherapy	Regorafenib + Nivolumab	50	10%	4,3	11,1	NA	ORR liver lesions: 4%; without liver lesions: 25%
NCT04126733	2023	2	≥ 2 previous lines of chemotherapy	Regorafenib + Nivolumab	70	7%	1,8	11,9	NA	ORR liver lesions: 0%; Grade 5 AEs in 3%
NCT04362839/RIN	2023	1	≥ 2 previous lines of chemotherapy	Regorafenib + Nivolumab + Ipilimumab	39	28%	4	20	NA	greater benefit for pts without liver mets
NCT03657641	2022	1/2	≥ 1 prior line of treatment	Regorafenib + Pembrolizumab	73	0%	2	10.9	NA	Largest trial of ICI + regorafenib in MSS CRC
NCT03475953/REGOMUNE	2022	2	≥ 1 prior line of treatment	Regorafenib + Avelumab	48	0%	3,6	10.8	NA	Better survival with increased CD8 + T-cell infiltration
NCT04695470	2022	2	≥ 2 previous lines of chemotherapy	Fruquintinib + Sintilimab	10	16%	4,1	13.3	NA	Grade 3 AEs in 90%
ChiCTR2000028965	2022	2	≥ 2 previous lines of chemotherapy	Fruquintinib + Toripalimab	30	0,17	6	8.0	NA	25% Grade 3–4 TRAE was reported
NCT03912857	2020	2	≥ 2 previous lines of chemotherapy	SHR-1210 (anti-PD-1 antibody) + Apatinib	2	0%	1,83	7,8	NA	high grade of AEs
NCT03539822/CAMILLA	2024	1/2	≥ 2 prior line of treatment	Cabozantinib + Durvalumab	29	27,6%	4,7	9,1	NA	ORR was 41.67% in CPS ≥ 5 patients
NCT03797326/LEAP-005	2021	2	≥ 2 previous lines of chemotherapy	Lenvatinib + Pembrolizumab	32	22%	2,3	7,5	NA	
NCT02788279/IMblaze370	2019	3	≥ 2 previous lines of chemotherapy	Atezolizumab + Cobimetinib	183	3%	1,9	8,9	PFS: HR 1,25 OS: HR 1,00 p = 0,99 vs Regorfenib	
NCT03170960/COSMIC-021	2022	1	First line	Cabozantinib + Atezolizumab	31	9,7%%	3	14	NA	greater benefit for RAS WT pts
			**IO ± IO**							
NCT00730639	2012	1	≥ 2 previous lines of chemotherapy	Nivolumab	7	0	NA	NA	NA	
NCT01876511/KEYNOTE-016	2015	2	≥ 2 previous lines of chemotherapy	Pembrolizumab	18	0%	2,2	5	NA	
NCT02788279/IMblaze370	2019	3	≥ 2 previous lines of chemotherapy	Atezolizumab	90	2%	1,9	7,1	PFS: HR 1,39 OS: HR 1,19; p = 0,34 vs Regorfenib	
NCT03642067	2024	2	≥ 1 prior line of treatment	Nivolumab + Relatlimab	59	5,1%	2,2–2,6	4,3–14,2	NA	clinical benefit rate 50% in lung only
NCT03860272/C-800	2023	1	≥ 1 prior line of treatment	Botensilimab + Balstilimab	70	30%	4,1	NR or 9,4	NA	ORR liver lesions: 0%OS: 9,4 m, ORR in patients without liver mets: 23%,OS:NR
NCT02870920/CCTG CO.26	2020	2	multiple prior lines of chemotherapy	Tremelimumab + durvalumab	119	1,2%	1,8	6,6	PFS: HR 1,01; p = 0,97 OS: HR 0,72; p = 0,07 vs BSC	TMB > 28/Mb (21%) had OS benefit (HR: 0.34, 90% CI: 0.18–0.63); G3-4 AEs in 64%
NCT02060188/CheckMate 142	2022	2	≥ 1 prior line of treatment	Nivolumab + Ipilimumab	23	NA	1,4	NA	NA	

### Immune Checkpoint Inhibitors in Advanced pMMR/MSS mCRC: Monotherapy or Combination Therapy?

The absence of antitumor activity for single-agent ICI in refractory pMMR/MSS mCRC has been substantiated in multiple clinical trials involving nivolumab, pembrolizumab, and the anti-PDL1 inhibitor atezolizumab. For instance, an early pan-tumor basket trial investigating the efficacies of pembrolizumab as well as nivolumab demonstrated no meaningful response rates in pMMR/MSS mCRC patients [7, 14]. Similarly, atezolizumab in comparison with the multi-kinase inhibitor regorafenib failed to show any efficacy in these patients [15]. These unsatisfactory results prompted the investigation of combination therapies. Combination therapies are intended to ensure that chemotherapy strengthens the immune response by increasing the immunogenicity of cancer cells or reducing immunosuppressive mechanisms. Important checkpoints in CRC are lymphocyte-activation gene 3 (LAG3), CTLA4, T-cell immunoglobulin and mucin-domain containing-3 (TIM-3) and PDL1, which control tumor growth and progression [16].

The METIMMOX study evaluated the efficacy between FLOX with and without nivolumab in untreated unresectable MSS mCRC. Unfortunately, the addition of nivolumab did not show a significant advantage in terms of PFS. However, in the subgroup of ≥ 60 years patients, the addition of nivolumab showed a significant survival improvement (PFS 13.6 months, p = 0.021), which suggests a potential benefit in this subgroup [17]. The combination of CTLA4 inhibitors with PDL1 inhibitors was investigated in the single arm study MEDTREME [18]. The combination of durvalumab and tremelimumab in combination with mFOLFOX6 was evaluated in *RAS*-mutated unresectable mCRCs. They set the hypothesis that the FOLFOX regime could trigger immunogenic cell death and eliminate myeloid-derived suppressor cells. The trial showed promising efficacy, with a PFS prolongation of 8.2 months [19]. Additional studies, such as MAIA [20], used the combination of botensilimab (CTLA4 inhibitor) and balstilimab (PDL1 inhibitor), which has shown promising effects in improving T-cell function. With an ORR of 17%, a DCR of 61% and a 12-month OS rate of 60%, the combination therapy has potential efficacy in pMMR/MSS mCRC patients [21]. These combinations show promising results in pMMR/MSS mCRC mCRCs, especially in the subpopulation without liver metastases. Other still ongoing studies which were presented at the major oncology conferences in 2023/24 regarding immunotherapy in pMMR/MSS mCRC are summarised in Table 1.

### Immune Checkpoint Inhibitors Combined with Tyrosine-Kinase Inhibitors

Targeted therapies selectively influence molecules involved in cancer cell proliferation, growth and metastasis [22]. Multiple trials have been conducted so far to investigate the optimal combination partners with ICIs. A single-center non-randomized clinical trial [23] (NCT04362839) evaluated the combination of regorafenib, ipilimumab, and nivolumab in MSS mCRC. The RP2D cohort demonstrated an ORR of 36%, DCR of 68% and a PFS of 5.0 months in patients without liver metastases [24]. In the REGONIVO study [25], the combination of regorafenib and nivolumab showed promising data in the 1b trial with then lacking a confirmation of response in the phase 2 CRC only trial [26]. However, the combination of regorafenib with nivolumab increased the response rates and has shown to be able to overcome resistance to ICIs. In the presence of liver metastases, there are some restrictions, such as the lack of synergistic effect and the lack of predictive biomarkers. This treatment could represent a valid therapeutic option for patients with CRC without liver metastases [27].

In *BRAF*^*V600E*^-mutated and MSS mCRCs, a phase I trial [28] investigating the efficacy of cetuximab, camrelizumab, and vemurafenib (VCC) showed an ORR of 40.0% and a DCR of 80.0%. This suggests that VCC holds promise as a treatment option for this specific patient population justifying further investigation [29].

MEK inhibition in preclinical models increased MHC-I expression and the presence of antigen-specific CD8^+^ T cells in CRC. Combining MEK with PDL1 inhibitors resulted in tumor regression in mouse models in *KRAS*-mutated CRC [30]. On this basis, the phase III STELLAR-303 [31] study compares zanzalintinib, atezolizumab with regorafenib in mCRC patients with MSS/low-MSI and without liver metastases. Recruitment is still ongoing [32]. Additionally, one of the two randomized trials combining atezolizumab and cobimetinib, a MEK inhibitor, versus regorafenib monotherapy did not show any significant efficacy (IMBlaze370) [15]. Similarly, the LEAP trial, evaluating the multi-kinase inhibitor lenvatinib combined with pembrolizumab failed to demonstrate superior OS compared to standard-of-care chemotherapy in refractory advanced pMMR/MSS mCRC [33].

### Immune Checkpoint Inhibitors Combined with Chemotherapy

The combination of chemotherapy and ICI in pMMR/MSS mCRC has yielded conflicting results so far. In later lines of treatment, adding ICI to standard chemotherapy with or without vascular endothelial growth factor (VEGF) inhibitors did not reveal ground-breaking outcomes [34–36]. In contrast, studies evaluating VEGF inhibitors and ICIs in the first-line setting showed promising ORR and survival data. The phase II AtezoTRIBE trial compared FOLFOXIRI plus the VEGF antibody bevacizumab with or without atezolizumab and reported a significant improvement in PFS [37]. A single-arm phase I/II study evaluated the addition of the anti-PDL1 inhibitor durvalumab and the anti-CTLA4 inhibitor tremelimumab to first-line FOLFOX regimen in patients with *RAS*-mutated mCRC observing a PFS of 8.2 months and an ORR of 63% compared to historical controls of FOLFOX without bevacizumab (PFS: 5–6 months; ORR: 36%) [19]. Moreover, the CheckMate 9X8 trial investigated mFOLFOX6 plus bevacizumab combined with nivolumab in the first line, showing higher benefits in the subgroup of TMB-high patients [38].

Despite the lack of trailblazing OS improvements in these combination strategies, new therapeutic approaches are eagerly awaited. A potential near-future practice-changing approach was recently presented at the annual meeting of the American Society of Clinical Oncology (ASCO) in 2024. The ARC-9 phase 2 trial combined etrumadenant, a novel adenosine receptor inhibitor, with zimberelimab, another anti-PD1 antibody, and standard re-challenge FOLFOX plus bevacizumab therapeutics. This combination yielded an ORR of 13%, a PFS of 6.2 months, and an OS of 19.7 months significantly outperforming the control arm with regorafenib. Comparing this mostly third-line population in a cross-trial manner to the new standard third-line treatment of trifluridine/tipiracil plus bevacizumab, the ARC-9 trial reported the most promising survival data in the third-line setting to date [39].

### Immunotherapy in Combination with Monoclonal Antibodies

#### Immunotherapy Plus Anti-EGFR Antibodies

Previous evidence suggests that anti-epidermal growth factor receptor (EGFR) therapies may enhance tumor response and trigger an immunogenic apoptosis cascade that is usually associated with increased expression of CTLA4 and PDL1 [40]. AVETRIC [41], a phase 2 study has investigated the addition of avelumab (PDL1 inhibitor) to the anti-EGFR antibody cetuximab and FOLFOXIRI in the first-line treatment of pMMR/MSS mCRC that do not harbour any mutation in pan-*RAS* or *BRAF* genes. It met its primary endpoint and showed promising results with a PFS of 14.1 months and an ORR of 82% [42].

#### Immunotherapy Plus Antiangiogenic Antibodies

Several studies have shown that antiangiogenic agents, by inhibiting VEGF signaling, normalize tumor vasculature and increase T-cell infiltration, thereby enhancing immune cell activation [43]. Atezolizumab is currently approved for the treatment of metastatic non-small cell lung cancer and advanced urothelial carcinoma [44]. The Atezo-TRIBE [45] study, as already mentioned above, included 218 patients of whom were 92% MSS. In this subgroup, the addition of atezolizumab to bevacizumab and chemotherapy demonstrated an increase in 37 months-PFS (11.5 months (dual) vs 13.0 months (triple)). Furthermore, patients in this trial with a high immunoscore (CD3^+^ and CD8^+^ T cells) and/or immunoscore-IC (CD8^+^ and PDL1^+^) showed an improved PFS compared to those in their respective low group. Interestingly, the amount of tumor infiltrating lymphocytes correlated poorly with the immunoscore indicating that the presence of special subpopulations of T cells contributes to ICI response [46]. These results show that patients with elevated Immunoscore IC and/or TMB in pMMR/MSS mCRC experience a survival benefit when atezolizumab was added. The combination of chemotherapy and immunotherapy has not yet achieved clinically meaningful results in pMMR/MSS mCRC patients. Further trials are still ongoing.

#### Immunotherapy in Combination with Radiotherapy

Up to two-thirds of all cancer patients with solid tumors undergo radiation therapy underscoring its pivotal role as a standard-of-care treatment modality. The primary objective of radiotherapy is to achieve local tumor control by inducing DNA damage, resulting in cell death and subsequently enhancing the immune system’s anti-tumor response [47]. Combining radiotherapy with either one or multiple ICIs or with anti-VEGF targeted therapies, may be a promising approach overcoming primary resistance upon ICI.

The FRUIT trial [48] investigated fruquintinib combined with the IgG4 antibody tislelizumab and SBRT in later-line therapy in pMMR/MSS mCRC. It showed a PFS of 5.1 months and among 24 enrolled patients, six achieving partial response and 13 stable disease, resulting in an ORR of 26% and a DCR of 83% [49]. Furthermore, the NEST-1 trial tested the safety and efficacy of neoadjuvant botensilimab and balstilimab in patients with pMMR/dMMR CRCs showing significant tumor regression without delaying surgery or increasing severe adverse events. The positive results suggest that this regimen could potentially downstage tumors reducing the need for surgery and/or adjuvant chemotherapy independent of MMR/MSS status [50]. Currently, a further study has been initiated to further explore dosing and timing of this ICI combination [51].

#### Immunotherapy in Combination with Vaccine

One treatment method that has garnered considerable interest in recent years is cancer vaccine therapy. These vaccines target individual peptides of antigens released by tumor cells and trigger the process of activating the immune response [52]. Certain cytotoxic drugs have been described to have immunomodulatory effects and may act synergistically with cancer vaccines by enhancing their antitumor activity. Recently, nano- and neoantigen vaccines have emerged as a potential strategy to overcome the resistance upon ICI [53]. However, if this approach will enter clinical practice remains to be elucidated.

## Conclusion

While ICI monotherapies show only limited efficacy, innovative combinations with targeted therapies, chemotherapy and radiotherapy are possible combinations in patients with pMMR/MSS mCRC. So far, the most promising results have been achieved by combining different ICIs, which deepen the responses when combined with radiotherapy, especially in localized rectal cancer. Nevertheless, further modulation of the tumor microenvironment (TME) and the identification of predictive biomarkers will be crucial for future developments which will be addressed in the next chapter.

### Biomarkers for Patient Selection

While previous sections have been focused on methods how to transform therapeutically „cold “ in „hot “ tumors, in this section we are focusing on biomarkers that have been evaluated to provide additional guidance to find a subset of pMMR/MSS mCRC patients who might benefit from ICIs. Despite these efforts, no clear conclusions can be drawn based on the data currently available. Herein, we are summarizing the most popular clinical and molecular biomarkers predicting ICI response.

### Programmed-Death Ligand 1 Expression

Programmed-death ligand 1 (PDL1) expression has been extensively evaluated in CRC using immunohistochemistry (IHC) [54–56]. The prevalence of PDL1 IHC expression in CRC varies significantly [56]. Although, PDL1 IHC expression is a routinely used biomarker for predicting ICI response for multiple other malignancies, such as lung or gastric cancer, this has not been the case for CRC, as demonstrated for instance in sub-analysis of the CheckMate-9 × 8 trial [38]. For this reason, PDL1 IHC status is currently not sufficiently validated to predict ICI response in mCRC patients. Therefore, additional predictive biomarkers are needed to select the right patient who might derive the greatest benefit from ICI.

### Tumor Mutational Burden

Tumor mutational burden (TMB) may serve as a viable metric to assess damage on a genomic level and may have implications for choice of treatment regimen. About 3% of all patients with CRC who harbour a MSS status, have a high-TMB. Definitions of high-TMB vary widely, from > 9 to > 37 mut/Mb, complicating comparisons across trials and tumor entities [57–59]. To facilitate comparisons between studies, experts and stakeholders defined a high-TMB tumor when TMB levels are > 10 mut/Mb [60]. Pembrolizumab, also FDA-approved for the treatment of high-TMB tumors, may be a good option in high-TMB mCRCs, irrespective of MSS status [61].

### DNA Polymerase Epsilon/Delta1 Mutations

*DNA polymerase epsilon/delta1 (POLE/POLD1)* mutations have shown to be associated with an improved outcome upon ICI in various tumor entities [62]. These mutations occur in about 1% of pMMR/MSS CRC patients [63] causing, regardless of the MSS status, hypermutations due to defects in the nucleotide and base excision repair system [64]. Wang et al*.* investigated different types of solid tumors treated with ICI with a special emphasis on *POLE* and *POLD1* mutations, wherein 75% of patients were pMMR/MSS. While *POLE/POLD1* mutations were detectable in around 2–3% in all malignancies, the prevalence was higher in CRC with a mutational rate found close to 7%. Moreover, their results revealed a significant survival benefit for ICI-treated patients; more than doubling the OS when comparing them to their non-mutated counterparts [65]. Only very recently, in a retrospective study, investigators analyzed data from 27 mCRC patients harboring *POLE/POLD1* mutations who were treated with ICI and compared them with a historical dMMR/MSI-H cohort [66]. The study revealed exceptional responses in terms of ORR (89%), PFS (HR: 0.17) and OS (HR: 0.24) for patients with *POLE/POLD1* mutations compared to dMMR/MSI-H patients. For this reason, *POLE/POLD1* mutations represent novel biomarkers predicting response to ICI.

### Consensus Molecular Subtypes

The consensus molecular subtypes (CMS) for CRC, described in 2015, define four clusters based on molecular signatures [67]. Briefly, CMS1 (MSI immune) summarizes tumors with dMMR/MSI-H, high CIMP, hypermutation, *BRAF* mutation and immune infiltration/activation. CMS2 (canonical) shows WNT & MYC activation and chromosomal instability (somatic copy number alterations (SNCA). CMS3 (metabolic) harbour also dMMR/MSI-H, but with low CIMP and *KRAS* mutations. CMS4 (mesenchymal) tumors have a high SNCA burden, stromal activation, TGF-β-activation and promotion of angiogenesis [67].

The Checkmate 9X8 trial revealed that adding nivolumab to standard care in pMMR/MSS mCRC patients is able to extend PFS, particularly in CMS1 and CMS3 patients and those with *KRAS*^*G12D/V/C*^ mutations [38]. How *KRAS* mutations are involved in the tumor immune microenvironment and in the sensitivity/resistance of ICI is currently the focus of research. The REGONIVO trial investigated the efficacy of regorafenib plus nivolumab in mCRC showing higher PFS in the CMS4 subgroup [27]. Responders showed gene-set enrichments in epithelial-mesenchymal transition pathways. IHC analyses revealed that responders correlated with higher infiltrations of CD8^+^ T cells, Tregs and M2 macrophages, thus constituting a “hotter” tumor microenvironment [68].

### Assessing the Tumor Microenvironment

ICI efficacy is heavily dependent on the activity of the patient’s immune system. It follows that the immediate tumor microenvironment is of interest for judging susceptibility to ICI, especially in the context of pMMR/MSS. Besides the AtezoTribe [37], the CAMILLA trial investigated cabozantinib with durvalumab in chemorefractory gastrointestinal cancer and showed similar findings using a transcriptomic approach. Gene-set variance analysis revealed increased interferon-γ signaling activity, IL-1 and IL-6 expression and T cell activity in patients who has shown a response to treatment. Moreover, differential gene expressions in non-responders and responders showing upregulation of genes associated with protein processing and changes in peptidase activity in responders, potentially affecting antigen presentation/processing [69]. These findings illustrate that characterizing the TME through different methods like DGE and subsequent GSVA could have potential impact on therapeutic choices, despite their limited availability and affordability.

### Impact of Liver Metastases

One putative negative “biomarker” whose impact has been shown is the comparative lack of efficacy of ICI in patients with liver metastasis. In a trial by Chongkai Wang et al., the ORR was approximately 20% in patients without liver metastases versus approximately 2% in those with liver metastases. Median PFS was 4.0 vs. 1.5 months for patients without versus with liver metastasis. These data suggest that mCRC MSS patients with liver metastases may benefit less from ICI therapy [70]. Data from another trial confirmed this data [21]. The investigators could show higher responses in mCRC patients without liver metastases when treated with an ICI combination. However, more prospective data are clearly necessary to corroborate these findings.

### Circulating Tumor DNA

Loree et al*.* used serial circulating tumor (ct)DNA to detect MAPK pathway alterations (SNV or indel in *RAS/EGFR/BRAF*^*V600*^ or MET or ERBB2 amplification) after various therapy lines. They could show that the exposure to anti-EGFR antibodies doubled the risk of new MAPK-alterations and increased the TMB, particularly in patients without existing MAPK alterations. By monitoring TMB with liquid biopsies, conclusions may be drawn about the efficacy of ICIs and patients who did not qualify initially might do so over the course of their treatment – i.e. making the tumor “hotter” by using anti-EGFR therapy. Applying this concept to monitor ICI using serial ctDNA might allow prediction of the development of secondary resistance mechanisms. Furthermore, anti-EGFR therapy induced TMB might contribute to making a tumor susceptible to ICI immunotherapy [71].

### Gut Microbiota

Microbiota alter response to ICI in various cancer types. One of the first cancers in which ICI has revolutionized the therapeutic management was in melanoma [72]. Routy et al*.* found that antibiotic therapy had an impact on the efficacy of PD1 inhibitors [73] and Vopalakrishnan et al*.* [74] showed that the gut microbiome composition influences therapeutic response and that a relative abudance of *Ruminococcaceae* bacteria may be advantageous.

Further studies [75, 76] showed that *Serratia*, *Cupriavidus* and *Sphingobium* species are increased in pMMR cancer patient’s microbiota. An additional study proposed a microbiota-based classification of CRC showing three subgroups with differences in survival rate and MSI status [77]. Despite the interaction between microbiota, cancerogenesis and treatment response, therapeutic consequences remain limited. Pre-clinical trials in mice with MSS CRC [78] showed that altering the gut microbiome using antibiotics blunted the response to anti-PD1 inhibitors, while *Lactobacillus adiophilus* lysates enhanced the efficacy of anti-CTLA4 inhibitors [79]. Various pre-clinical trials have shown that altering the gut microbiota in mice showed effects on ICI efficacy [80, 81].

### Cytokines

#### IL17A

IL17A, a cytokine, shows to be overexpressed in CRC [82] and has several effects on the TME. It induces the synthesis of PGEF2, which surpasses CXCL9/10 impeding infiltration of the TME by CD8^+^ T cells (see below.) [83]. Studies in mouse models of CRC [84] and cell lines [85] showed that IL-17 inhibits the recruitment of CD8^+^ T cells and increased PDL1 expression, thereby promoting resistance to anti-PD1 therapy. Therapeutic antibodies for blocking IL-17A have been developed for psoriasis and ankylosing spondylitis and one trial was registered combining secukinumab (anti-IL17) with camrelizumab (anti-PD1) therapy, but the current trial status is still unknown [86].

#### Chemokines

Chemokines, a subset of cytokines, interact with G-protein coupled receptors, inducing chemotaxis and contributing to extravasation [87]. Several chemokines can predict ICI response in CRC and other cancers. One chemokine axis involves CXCL9, -10 and -11 with a common downstream activation of CXCR3. Interferon gamma stimulates CXCL9 production [88] by monocytes, endothelial cells, fibroblasts and cancer cells leading to increased chemotaxis and infiltration of the TME [89]. CXCR3, the receptor of CXCL9/10/11 serves as a prognostic biomarker for CRC. Intratumoral activity of the CXCR3-chemokine system is necessary for anti-PD1 to work [90]. Meta-analysis has shown that high CXCL9 levels are associated with a good response to ICI in several cancers [91].

## Conclusion

Some biomarkers have been proposed for determining therapeutic susceptibility upon ICI in pMMR/MSS nCRC patients. Many represent surrogate markers of immune activity or immune cell infiltration of the TME. Some biomarkers, such as TMB or *POLE/POLD1* mutations, represent genotypes that are probably not inducible de-novo in pre-existing disease or that present distinct entities mimicking MSI, whereas some markers might be viable as a means of judging how successful efforts of making the TME “hot” have been.

### Future Perspective

Despite recent advances in identifying novel biomarkers and potential combinations of targeted therapies with chemotherapy or radiotherapy in MSS/pMMR mCRC, a definitive solution has not yet been established. The most promising approach so far involves radiotherapy combined with chemotherapy-free regimens, as highlighted by the NEST-1 trial, which demonstrate a higher likelihood of response to ICI in localized MSS/pMMR CRC with a low disease burden. Additionally, emerging strategies involving CAR-T cells, tumor vaccines, novel immune checkpoint inhibitors and biomarker stratified trials hold the potential to shape the future of oncology in this field.

## Data Availability

No datasets were generated or analysed during the current study.
